# Banding cytogenetics of the vulnerable species Houbara bustard (Otidiformes) and comparative analysis with the Domestic fowl

**DOI:** 10.3897/CompCytogen.v13i1.30660

**Published:** 2019-01-14

**Authors:** Leila Mahiddine-Aoudjit, Ouahida Boucekkine, Kafia Ladjali-Mohammedi

**Affiliations:** 1 University of Sciences and Technology Houari Boumediene (USTHB), Faculty of Biological Sciences, Laboratory of Cellular and Molecular Biology, Team of Developmental Genetics, PO box 32 El-Alia, Bab-Ezzouar, 16110 Algiers, Algeria University of Sciences and Technology Houari Boumediene Algiers Algeria; 2 University of M’hamed Bougara of Boumerdes, Faculty of Sciences, Department of Biology, Avenue de l’Indépendance, 35 000 Boumerdès, Algeria University of M’hamed Bougara of Boumerdes Boumerdès Algeria; 3 The General Direction of Forests, Ben Aknoun, Algiers, Algeria The General Direction of Forests Algiers Algeria

**Keywords:** *
Chlamydotis undulata undulata
*, endangered species, GTG- and RBG- banded karyotypes, interspecific comparison

## Abstract

The Houbara bustard *Chlamydotisundulata* (Jacquin, 1784) is an emblematic and endangered bird of steppes and desert spaces of North Africa. This species belonging to Otidiformes is recognized as vulnerable by the International Union for Nature Conservation.

The critical situation of this species and the revision of its classification on the tree of birds encouraged the authors to start accumulating chromosome data. For that, we propose the GTG- and RBG-banded karyotypes of the Houbara bustard prepared from primary fibroblast cell cultures. The first eight autosomal pairs and sex chromosomes have been described and compared to those of the domestic fowl *Gallusdomesticus* (Linnaeus, 1758). The diploid number has been estimated as 78 chromosomes with 8 macrochromosomes pairs and 30 microchromosomes pairs, attesting of the stability of chromosome number in avian karyotypes.

The description of the karyotype of the Houbara is of crucial importance for the management of the reproduction of this species in captivity. It can be used as a reference in the detection of chromosomal abnormalities, which would be responsible of the early embryonic mortalities.

## Introduction

With approximately 10,699 species, birds represent the class of Tetrapoda with the highest number of species (http://www.worldbirdnames.org). This class presents a certain number of particularities such as the presence of feathers, flight and a small genome (about 1.45 pg that represents 1/3 of the mammalian genome). As well, the avian karyotypes are very particular, with a very consistent diploid number. The range of variation is very wide, between 40 and 138 chromosomes, the average being from 76 to 82 for most species (Takagi and Sasaki 1974, De Boer 1976, De Boer and Sinoo 1984, Christidis 1990). About 18–23% of the avian genome is represented by microchromosomes (Smith and Burt 1998). However, they contain more than 50% of the genes (Smith et al. 2000), they are GC-rich (Auer et al. 1987) and enriched for CpG islands (McQueen et al. 1996). Moreover, the female represents the heterogametic sex named ZW and the male the homogametic sex ZZ (Christidis 1990).

Despite the diploid number that seems to be stable in birds, the avian genome has undergone multiple evolutionary events. Chromosome fission has previously been reported as being a factor of evolutionary change (Takagi and Sasaki 1974, Tegelström et al. 1983, Perry et al. 2004). Nevertheless, other regions of the genome can be subject to frequent breakage (Skinner and Griffin 2012). The analysis of macrochromosomes of chicken, turkey and zebra finch has provided evidence that the presence of hotspots facilitates chromosomal rearrangements (Kretschmer et al. 2018a).

Besides, phylogenetic analysis of 48 bird species representing all Neoaves orders was conducted and the analysis identified a first divergence of the Neoaves into two independent lines named Passerea and Columbea, without forgetting the emergence of the new order Otidiformes to which the endangered species Houbara bustard is now affiliated (Jarvis et al. 2014). In fact, before this study, and for a long time, the Houbara bustard was affiliated to the order of Gruiformes whose classification has been revised (Wetmore 1960, Roselaar 1980, Cracraft 1981, Olson 1985, Sibley and Ahlquist 1990, Sibley et al. 1993, Houde et al. 1997, Livezey 1998, Fain and Houde 2004).

The Houbara bustard is an emblematic bird of the large steppe areas and desert spaces of North Africa and the Middle East (Heim de Balsac and Mayaud 1962). Two monophyletic sister groups of bustards are considered (Tobias et al. 2010, Del Hoyo et al. 2014). Firstly, the Asian Houbara bustard *Chlamydotismacqueenii* (Gray, 1832) is found in the east of Egypt, from the Arabian Peninsula and Pakistan to Central Asia. On the other hand, the North African Houbara bustard *Chlamydotisundulata* is subdivided into two subspecies: *Chlamydotisundulataundulata* (Jacquin, 1784) extending from Morocco and northern Mauritania through Algeria, to the west of Egypt, and *Chlamydotisundulatafuerteventurae* (Rothschild & Hartert, 1894) which is endemic to the Canary Islands. Over the past 30 years, illegal harvesting of bustards and degradation of their environment has increased throughout its range. This has led to significant population decline in Africa and in Asia (Le Cuziat et al. 2005, Azafzaf et al. 2005). Therefore, the International Union for the Conservation of Nature has listed this species on the Red List as vulnerable (http://www.iucnredlist.org).

No description of the karyotype of the Houbara bustard has been reported to date. The only known cytogenetic data are a metaphase of this species, without a precise description of the chromosomes, which was reported in the study that allowed the development of chromosome paints and BACs for the characterization of inter- and intrachromosomal rearrangements of avian microchromosomes (Lithgow et al. 2014). However, several molecular studies based on the characterisation of microsatellites have been conducted in the Houbara bustard for the genotyping of individuals (Chbel et al. 2002, Pitra et al. 2004, Arif et al. 2012).

Here we describe the macrochromosomes of the Houbara bustard in morphologic GTG bands and dynamic RBG bands. Morphometric measurements were used to facilitate the classification of smaller macrochromosomes. The obtained banding pattern in Houbara bustard chromosomes was compared with that of the chicken chromosomes, in order to determine the presence of chromosomal rearrangements that would have occurred during speciation.

## Material and methods

### Embryos

Fifteen Houbara bustard embryos aged between 8 and 19 days were collected from Emirati Bird Breeding Centre for Conservation EBBCC (32°55'40.54"N, 0°32'33.71"E) in the region of Abiod Sidi Cheikh (Wilaya d’El-Bayadh, south of Algeria) during the breeding season. The embryos were obtained in accordance with the authorization from the General Direction of Forests of Algeria (N°30BOG/N°80DPFF/DGF-18).

### Cell culture and chromosomes preparations

Fibroblasts were isolated from Houbara bustard embryos by trypsinisation (trypsin solution 0.05%, Sigma) and incubated at 41 °C with RPMI 1640 culture medium (20 mM HEPES, Gibco) supplemented with 10% of fetal calf serum (FCS) (Gibco), 1% L-Glutamine (Sigma), 1% penicillin, streptomycin and fungizone (Sigma). Cultures of fibroblasts were synchronised as described by Ladjali et al. (1995), using a double thymidine block during S phase in order to increase the yield of metaphase and early metaphase cells. The 5-bromo-2’-deoxyuridine (BrdU) (final concentration: 10 μg/ml, Sigma) was added to prepare chromosomes to the RBG staining (Zakharov and Egolina 1968, Ladjali et al. 1995).

As a sufficient number of refractive mitotic cells was observed (after 6–8 h), they were treated with colchicine (final concentration: 0.05 μg/ml, Sigma) for 5 min at 37 °C. Cells were harvested by the addition of 0.05% trypsin-EDTA (Gibco). Hypotonic treatment was performed. In fact, cells were suspended for 13 min at 37 °C in hypotonic solution 1:5 (FCS- distilled water). Fixation and spreading were performed using standard methods (Dutrillaux and Couturier 1981, Ladjali et al. 1995).

### Staining procedures

The revelation of the structural GTG bands is based on enzymatic digestion with proteolysis (Seabright 1971, Ladjali et al. 1995). Aged (3–10 days) slides were incubated for 14 seconds in a fresh trypsin (Sigma) solution (final concentration: 0.25%) and stained for 10 min with 6% Giemsa (Fluka) solution (Ladjali et al. 1995).

The RBG-FPG staining (R-bands obtained with BrdU by Fluorochrome-photolysis and Giemsa staining) was performed as previously described (Romagnano and Richer 1984, Schmid et al. 1989, Viegas-Péquignot et al. 1989, Ladjali et al. 1995). The slides were incubated in a solution of Hoechst 33258 (final concentration: 0.01 mg/ml) during 20 min, followed by an incubation in fresh 2×SSC solution at a distance of 15 cm from blacklight blue (NARVA, LT18W/073) during 90 min. The slides were immersed in Earle’s buffer (pH= 6.5) at 87 °C for 10 minutes and stained with 6% Giemsa (Fluka) for 10 min.

To make a comparison with chromosomes of the chicken, GTG banding was also performed on previously frozen chicken chromosome preparations.

### Chromosome classification and measurement

Slides were analysed using Axio Scope A1 (Zeiss) and thirty metaphases with decondensed chromosomes were selected and photographed with CoolCube1 (Metasystems). Houbara bustard chromosomes were classified according to the International System of Standardised Avian Karyotypes (ISSAK) (Ladjali-Mohammedi et al. 1999).

The first eight pairs of chromosomes of the Houbara bustard and the domestic fowl and their sex chromosomes were measured using KARYOTYPE 2.0 software (Altinordu et al. 2016). The rest of the chromosomes were not measured because of their very small size. Different parameters of morphometry are presented: length of the long (*q*) and the short (*p*) arms, total length (p+q), arm ratio (r= q/p) and the centromeric index (CI% = p/p+q × 100).

## Results

**Figure 1.**
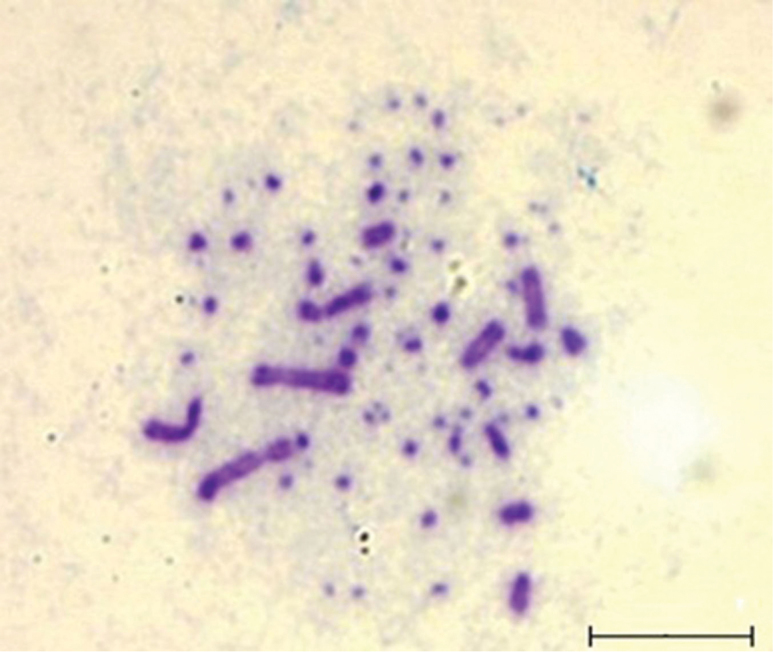
Metaphase of the Houbara bustard showing macrochromosomes and microchromosomes with Giemsa staining. Scale bar: 5 μm.

The diploid number of the Houbara bustard has been estimated as 78 chromosomes by examination of full metaphases (Fig. 1). Although the use of double synchronisation can produce decondensed chromosomes, classic cytogenetic techniques alone do not accurately count and describe microchromosomes. Often, they are dispersed outside the metaphases during spreading or hidden by other chromosomes (Tegelström and Ryttman 1981).

**Figure 2.**
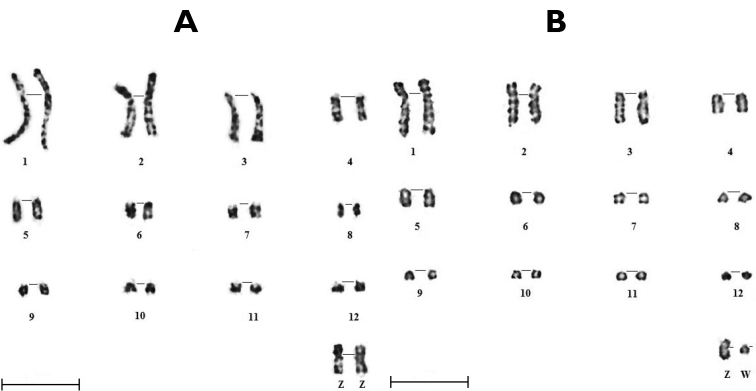
GTG **(A)** and RBG **(B)** karyotypes of the first 12 and sex chromosomes of Houbara bustard *Chlamydotisundulataundulata*. Scale bar: 5 μm.

**Figure 3.**
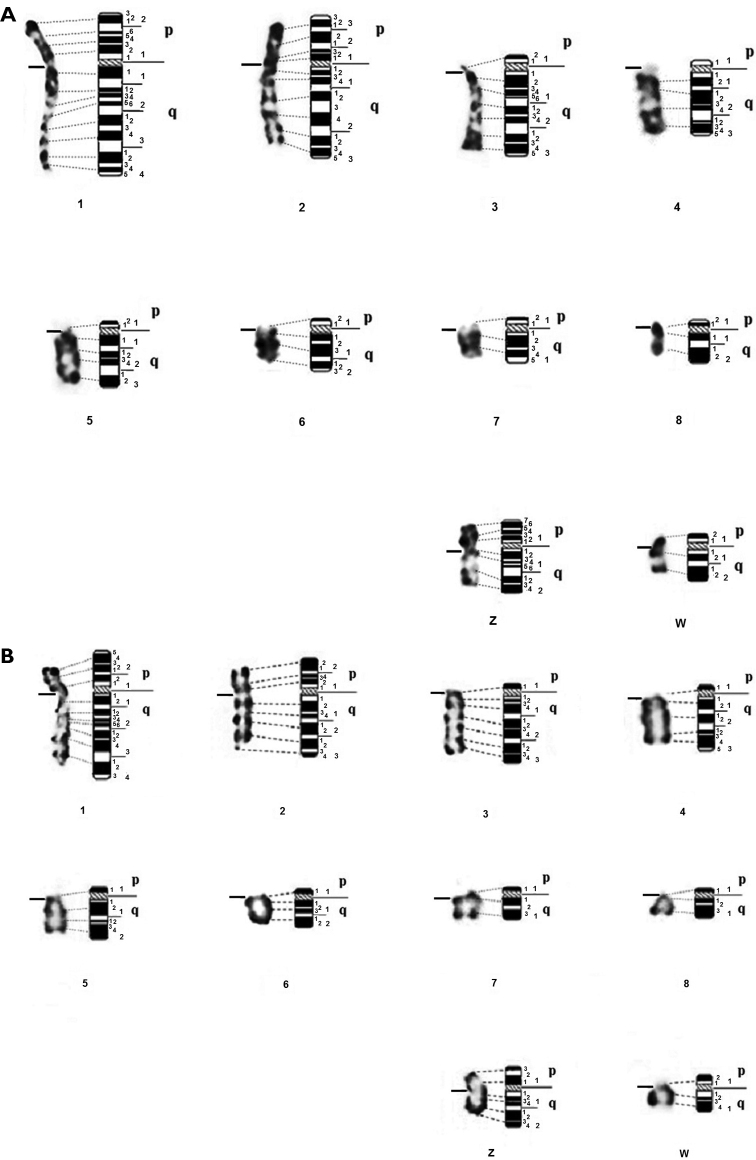
GTG-banded **(A)** and RBG-banded **(B)** macrochromosomes, sex chromosomes ZW and their corresponding ideograms of Houbara bustard *Chlamydotisundulataundulata*.

In this study, we propose the karyotype of the Houbara bustard with morphological GTG-banded chromosomes (Fig. 2A) and dynamic RBG-banded chromosomes (Fig. 2B). Partial ideograms of the Houbara bustard have been established (Fig. 3A, B) to describe precisely the chromosomes (Table 1).

**Table 1.** Description of GTG and RBG bands on macrochromosomes and sex chromosomes ZW of the Houbara bustard.

Chromosomes	GTG bands / ideograms	Description	RBG bands / ideograms	Description
**1**	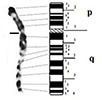	– **Short arm (p)**	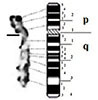	– **Short arm (p)**
**submetacentric**		2 regions		2 regions
		9 bands		7 bands
		– **Long arm (q)**		– **Long arm (q)**
		4 regions		4 regions
		16 bands		15 bands
**2**	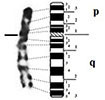	– **Short arm (p)**	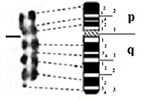	– **Short arm (p)**
**submetacentric**		3 regions		2 regions
		8 bands		6 bands
		– **Long arm (q)**		– **Long arm (q)**
		3 regions		3 regions
		13 bands		10 bands
**3**	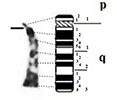	– **Short arm (p)**	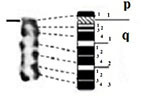	– **Short arm (p)**
**acrocentric**		1 region		1 region
		2 bands		1 band
		– **Long arm (q)**		– **Long arm (q)**
		3 regions		3 regions
		14 bands		12 bands
**4**	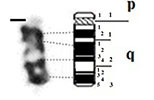	– **Short arm (p)**	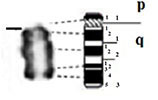	– **Short arm (p)**
**acrocentric**		1 region		1 region
		1 band		1 band
		– **Long arm (q)**		– **Long arm (q)**
		3 regions		3 regions
		11 bands		9 bands
**5**	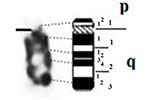	– **Short arm (p)**	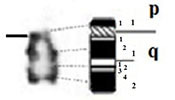	– **Short arm (p)**
**acrocentric**		1 regions		1 regions
		2 bands		1 band
		– **Long arm (q)**		– **Long arm (q)**
		3 regions		2 regions
		7 bands		6 bands
**6**	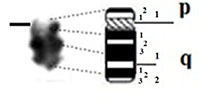	– **Short arm (p)**	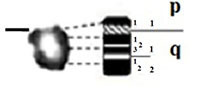	– **Short arm (p)**
**acrocentric**		1 region		1 region
		2 bands		1 band
		– **Long arm (q)**		– **Long arm (q)**
		2 regions		2 regions
		6 bands		5 bands
**7**	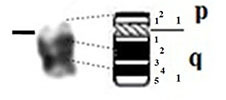	– **Short arm (p)**	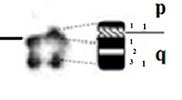	– **Short arm (p)**
**acrocentric**		1 region		1 region
		2 bands		1 band
		– **Long arm (q)**		– **Long arm (q)**
		1 region		1 region
		5 bands		3 bands
**8**	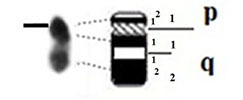	– **Short arm (p)**	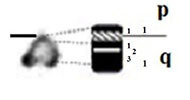	– **Short arm (p)**
**acrocentric**		1 region		1 region
		2 bands		1 band
		– **Long arm (q)**		– **Long arm (q)**
		2 regions		1 region
		3 bands		3 bands
**Z**	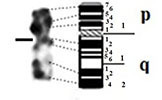	– **Short arm (p)**	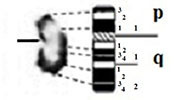	– **Short arm (p)**
**submetacentric**		1 region		1 region
		7 bands		3 bands
		– **Long arm (q)**		– **Long arm (q)**
		2 regions		2 regions
		10 bands		8 bands
**W**	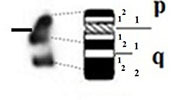	- **Short arm (p)**	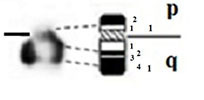	- **Short arm (p)**
**submetacentric**		1 regions		1 regions
		2 bands		2 bands
		- **Long arm (q)**		- **Long arm (q)**
		2 regions		2 regions
		4 bands		4 bands

The haploid karyotype of the first 10 autosomes and the sex chromosomes Z and W of the Houbara bustard corresponds to 130 GTG bands and 104 RBG bands. The number of bands obtained for this species is lower than that reported in the chicken for the same chromosome number (209 bands G and 182 bands R) (Ladjali et al. 1995).

In order to compare the chromosomes of the Houbara bustard and those of the domestic fowl, the first eight macrochromosomes and the sex chromosomes ZW of these two species have been measured (Table 2).

**Table 2.** Measurements of eight macrochromosomes and sex chromosomes ZW of Houbara bustard *Chlamydotisundulataundulata* and Domestic fowl *Gallusdomesticus*. **Chr**: chromosome, **q**: long arm, **p**: short arm, **p+q**: total length, **r**: arm ratio q/p, **CI** %: centromeric index=p/p+q × 100. Lengths are given in micrometer (µm) ± standard deviation.

**Chr**	**Houbara bustard**					**Domestic fowl**				
	**q**	**p**	**p+q**	**r**	**CI** %	**q**	**p**	**p+q**	**r**	**CI** %
**1**	2.93±0.57	1.19±0.29	4.12±0.81	2.46	29 %	6.69±1.26	3.97±0.70	10.66±1.85	1.69	37%
**2**	2.08±0.41	0.95±0.24	3.02±0.63	2.19	31 %	5.35±0.71	2.76±0.54	8.11±1.24	1.94	34%
**3**	2.15±0.47	0.12±0.05	2.26±0.49	18.50	5 %	5.42±0.66	0.36±0.05	5.77±0.62	15.18	6%
**4**	1.33±0.25	0.12±0.05	1.45±0.27	10.98	8 %	3.72±0.59	0.96±0.15	4.69±0.72	3.86	20%
**5**	1.21±0.20	0.09±0.04	1.29±0.22	13.37	7 %	2.71±0.43	0.29±0.12	3.00±0.51	9.39	9%
**6**	0.87±0.16	0.06±0.06	0.92±0.18	15.86	6 %	1.53±0.16	0.07±0.10	1.60±0.24	21.83	4%
**7**	0.79±0.11	0.02±0.03	0.77±0.12	41.89	2 %	1.28±0.23	0.40±0.05	1.68±0.28	3.18	24%
**8**	0.67±0.13	0.01±0.02	0.67±0.12	92.52	1 %	0.89±0.14	0.61±0.05	1.49±0.15	1.46	41%
**Z**	0.99±0.20	0.46±0.13	1.44±0.31	2.17	32 %	2.30±0.34	2.04±0.29	4.34±0.63	1.12	47%
**W**	0.59±0.15	0.20±0.07	0.78±0.17	3.01	25 %	0.95±0.35	0.60±0.28	1.55±0.60	1.59	39%

## Discussion

The diploid number has been estimated in the Houbara bustard as 78 chromosomes as in many birds. Indeed, the diploid number is highly conserved with about 63% of birds with a chromosome number that varies between 74 and 86 (Christidis 1990, Rodionov 1997). The relatively unchanged nature of the diploid number amongst the majority of avian species implies that the organisation of bird karyotypes is a highly successful means of genome organisation. The karyotype of the Houbara bustard belonging to the new order of Otidiformes (Del Hoyo et al. 2014) appears very similar to the ancestral karyotype of birds with 8 pairs of macrochromosomes and 30 pairs of microchromosomes.

The size of the first eight pairs of chromosomes of the Houbara bustard varies between 4 μm (chromosome 1) and 0.67 μm (chromosome 8). This average size of Houbara bustard macrochromosomes is lower to the estimated size (3 to 6 μm) for avian macrochromosomes (Rodionov 1996). A significant decrease in the size of the bustard chromosomes after the third pair has been noted (Table 2).

The comparison of the first eight pairs and sex chromosomes of the Houbara bustard with those of the chicken revealed the presence of similarities as well as differences between these two species. Indeed the karyotype of the chicken conserves the ancestral karyotype of many avian orders (Guttenbach et al. 2003, Shibusawa et al. 2004, Griffin et al. 2007).

The first three chromosomes of the Houbara bustard are morphologically similar to those of the chicken. Chromosome 1 and 2 are submetacentric and chromosome 3 is acrocentric. These results are in agreement with those of Takagi and Sasaki (1974), who showed the conservation of the first three chromosomes in nine different orders of birds and in different species belonging to the family of Gruidae (Order Gruiformes) known to be the family closest to Otididae (Belterman and De Boer 1984).

**Figure 4.**
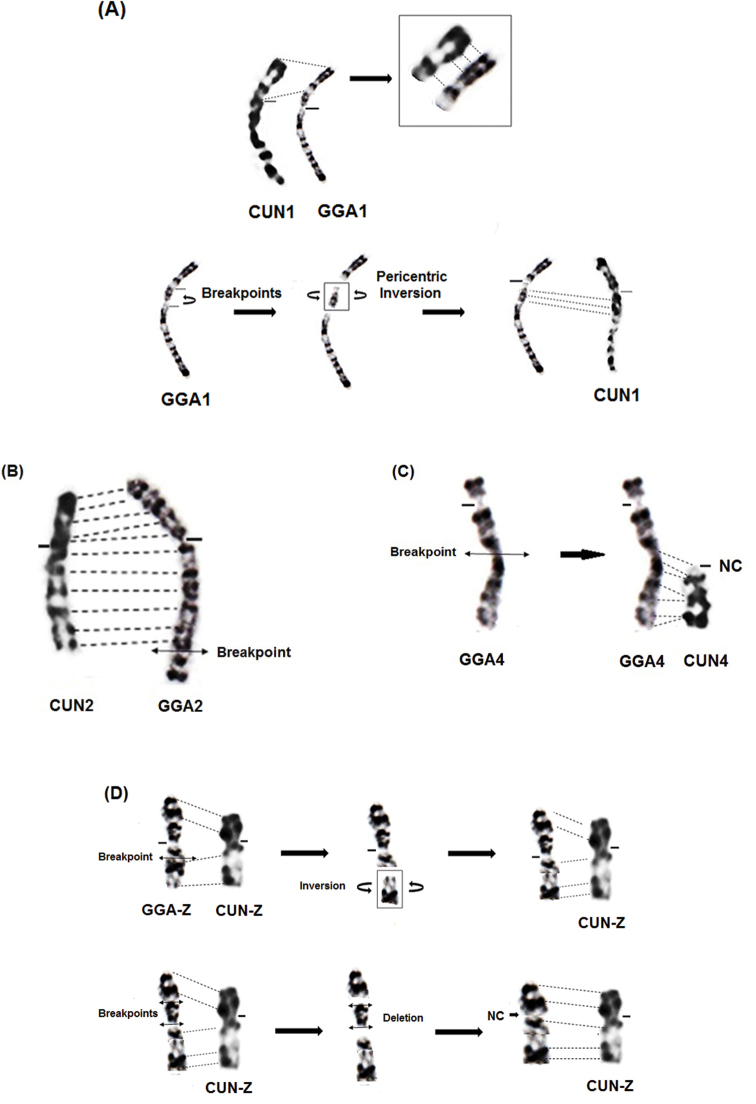
Representation of chromosomal rearrangements that could have occurred during the formation of the chromosome 1 **(A)**, the chromosome 2 (**B)**, the chromosome 4 **(C)** and the chromosome Z **(D)** of the Houbara bustard.

The comparison of the chromosome 1 of Houbara bustard with that of the chicken revealed a difference in the size of the p- arm of chromosome 1 (1p) which is shorter in the bustard (Fig. 4A). The arm ratio (q/p) is 2.46 in the chromosome 1 of the Houbara bustard while it is equal to 1.69 for that of the chicken. Furthermore, the comparison of GTG bands showed an inversion of patterns. The difference in the morphology of the chromosome 1 of Houbara bustard and the chicken, associated to the difference in GTG banding pattern in these two species, could be explained by a pericentric inversion that occurred in the chromosome 1 of the chicken, which is close to the ancestral chromosome 1 (Fig. 4A). This result must be confirmed by the use of molecular markers that will confirm this hypothesis and determine the extent of the rearrangement.

As for chromosomes 1 of Japanese quail and chicken, the high-resolution mapping using fluorescent *in situ* hybridisation with molecular markers on stretched chromosomes in the lampbrush showed that the difference in the morphology of this chromosome should be explained by de novo centromere formation and the hypothesis of centrometric inversion should be excluded (Zlotina et al. 2012).

Likewise, the long arm (q) of chromosome 2 of the Houbara bustard is shorter than that of the chicken and that would be the consequence of a terminal fission (Fig. 4B), and this distal part lost would eventually be involved in another independent event of chromosomal rearrangement (Furo et al. 2015). Because the bustard is phylogenetically distant from the domestic fowl, the different rearrangements are not visible (Prum et al 2015). The chromosome 3 is acrocentric and is apparently conserved in the two species.

The chromosome 4 of the Houbara bustard is acrocentric while that of the chicken is telocentric. Their arm ratios are equal to 10.98 and 3.86 respectively (Table 2). The comparison of the banding pattern reveals that chromosome 4 of the bustard corresponds to the terminal (q) arm of that of the chicken (Fig. 4C). This would be the consequence of a loss of the short arm (p) and a part of the long arm (q) during evolution. In fact, the hybridisation of chicken macrochromosomes on the metaphases of avian species from Anseriformes, Gruiformes and Passeriformes, revealed the hybridisation of the GGA4 on three different chromosomes in Gruiformes. The large region of GGA4 corresponds to the short arm of the metacentric chromosome of the coot FAT4 (*Fulicaatra*, Gruiformes) and the remaining part is found on two other chromosomes (FAT 7 and FAT 13) (Nanda et al. 2011).

The chromosome 5 of the Houbara bustard is acrocentric like that of the chicken but it seems to have lost the terminal part of the long arm. Indeed, chromosome 5 of the Houbara bustard measures 1.29 ± 0.22 µm and that of the chicken 3 ± 0.51 µm. This could be explained by a fission event that would have occurred during evolution. In fact, chromosome 5 of the chicken appears to be distributed on the short arm (p) of chromosome 4 of the coot (FAT4) and on microchromosome 12 (FAT12) (Nanda et al. 2011). The most noticeable is the association between GGA4 / GGA5 in this species of coot (*Fulicaatra)*, since chromosomes 4 and 5 of the chicken hybridized on the same FAT4 chromosome. This proves the presence of several fission and fusion events in Gruiformes (Nanda et al. 2011).

In contrast to chromosome 6 of the bustard which appears to be morphologically similar to that of the chicken, the chromosomes 7 and 8 of these two species are different. Houbara bustard chromosomes 7 and 8 are acrocentric whereas they are, respectively, telocentric and submetacentric in chicken (Ladjali-Mohammedi et al. 1999). Their arm ratios are, respectively, 41.89 and 92.52 in the bustard and equal to 3.18 and 1.46 in the chicken (Table 2). The morphological difference of these two chromosomes between the Houbara bustard and the chicken could be explained by the formation of neocentromere, or the occurrence of a pericentric inversion. The different suggestions for chromosomal rearrangements must be confirmed by molecular investigations in order to elucidate the phylogenetic relationship between the Houbara bustard and the Domestic fowl, as has already been done in other species.

Finally, the sex chromosome Z of the Houbara bustard differs from that of the chicken. It is submetacentric in the first species and metacentric in the second. The arm ratio (q/p) is 2.17 for the chromosome Z of the bustard while it is equal to 1.12 for that of the chicken (Table 2). In addition to the position of the centromere that is different in the chromosome Z of the chicken, we noted the loss of the p1.1 → p1.3 region corresponding to the chicken chromosome Z, as well as an inversion in the order of the GTG bands in the distal part of the long arm (Fig. 4D).

Chromosome Z that is metacentric in chicken appears to be submetacentric in many other species of Galliformes (Nanda et al. 2008). Also, a terminal inversion has been reported on chromosome Z of Chukar partridge (Ouchia-Benissad and Ladjali-Mohammedi 2018).

Despite the conservation of this chromosome in its totality during evolution, it appears to be subject to intrachromosomal rearrangements (Griffin et al. 2007, Nanda et al. 2008). This was confirmed by the inverted order of five orthologous genes (DMRT1, GHR, CHRNB3, ALDOB, B4GALT1) located on the chicken Z chromosome and mapped in eight other species (Nanda et al. 2008).

The W chromosome is submetacentric in the Houbara bustard. It appears to be morphologically similar to that of the chicken. Depending on its size, it can be classified between chromosome 6 and 7. The W chromosome in birds and reptiles seems to have degenerated during evolution. It is physically small, with a high proportion of heterochromatin (Ellegren 2011) that it is supposed to come from the accumulation of repetitive sequences and their conservation during evolution (Schartl et al. 2016).

In conclusion, this analysis of the chromosomes of the endangered Houbara bustard provided a precise description of a part of its karyotype in GTG and RBG bands. Chromosomal informations have been obtained for the newly established Otidiformes order. The identification of microchromosomes by fluorescence *in situ* hybridisation of specific BAC clones of chicken chromosomes is conceivable to complete the description of the karyotype of this species. The various rearrangements suggested must be confirmed by molecular studies of BAC clones localisation and chromosome painting for a better knowledge of avian karyotypes evolution.
